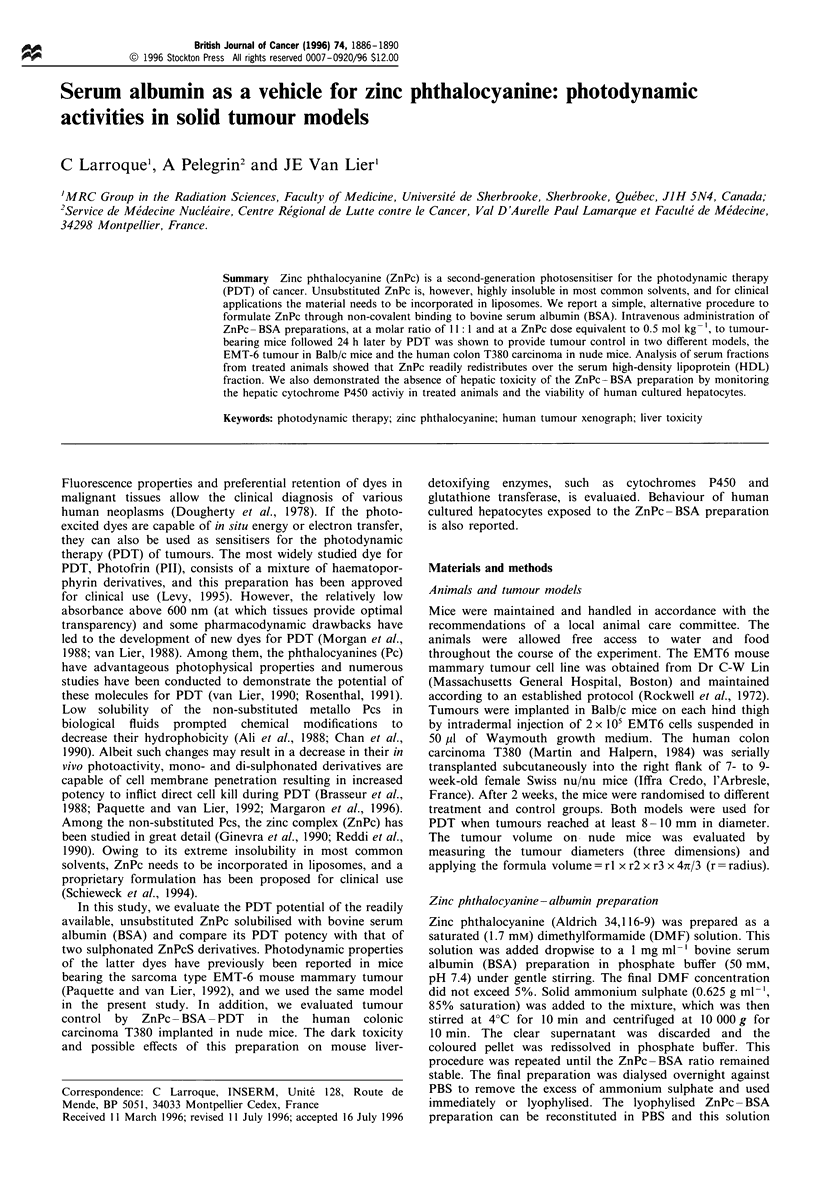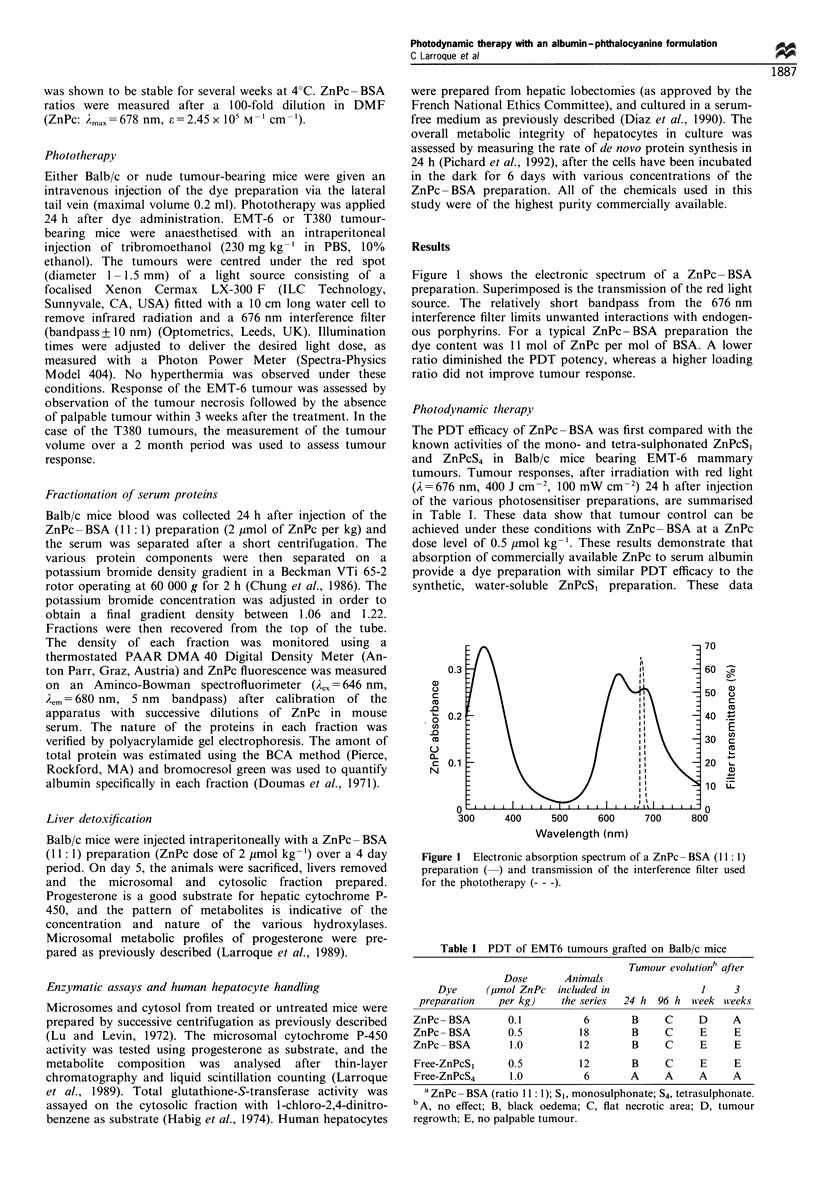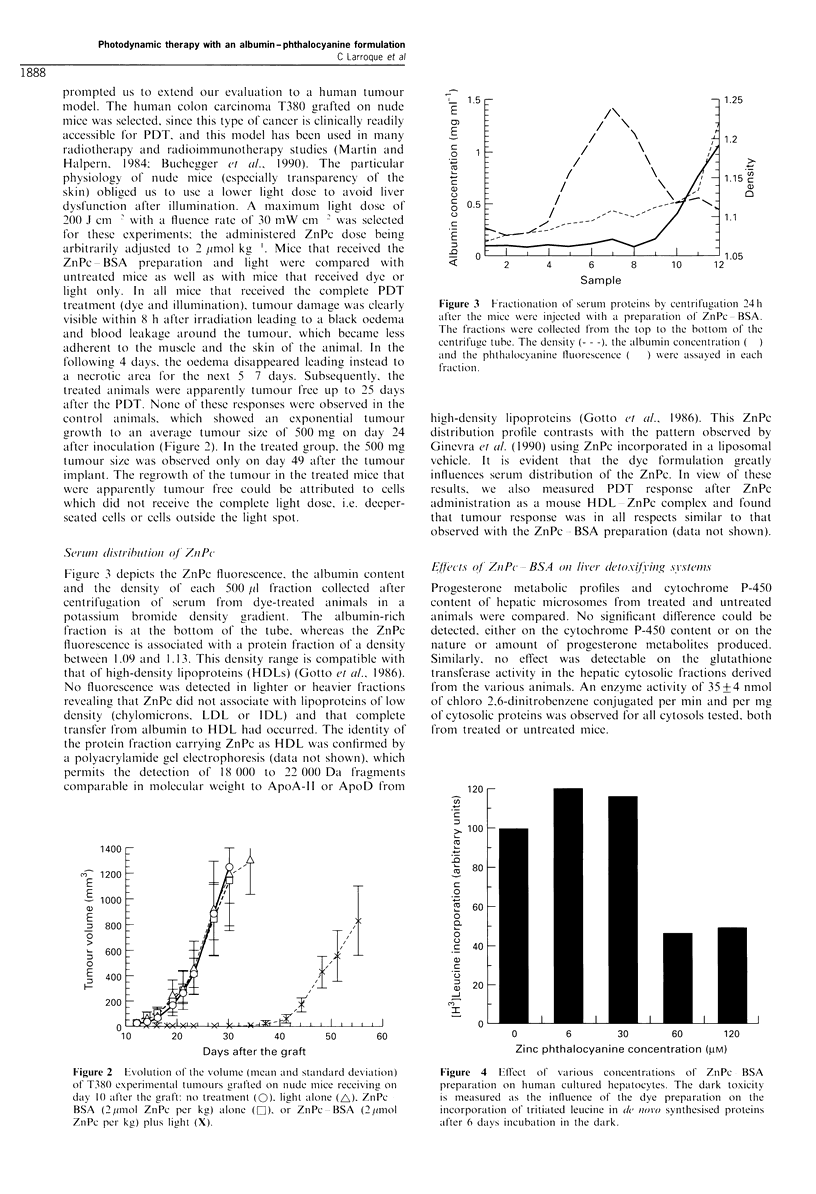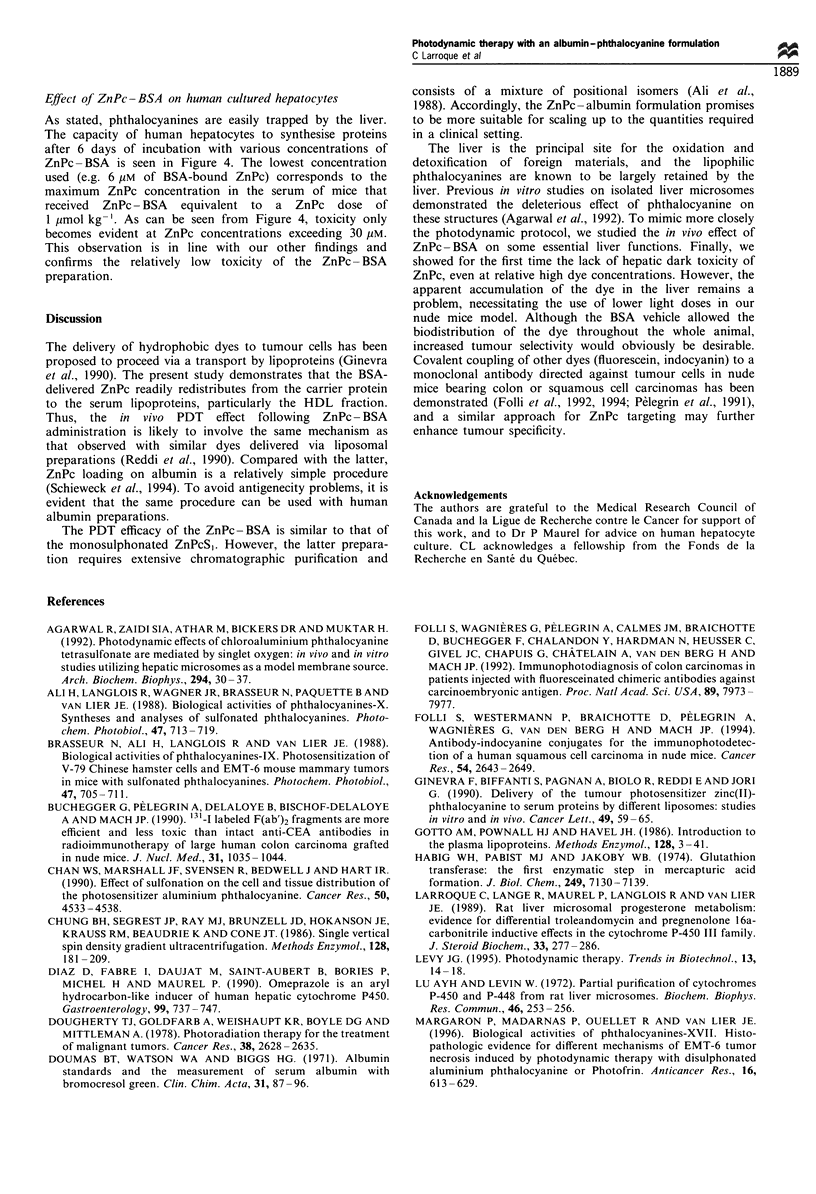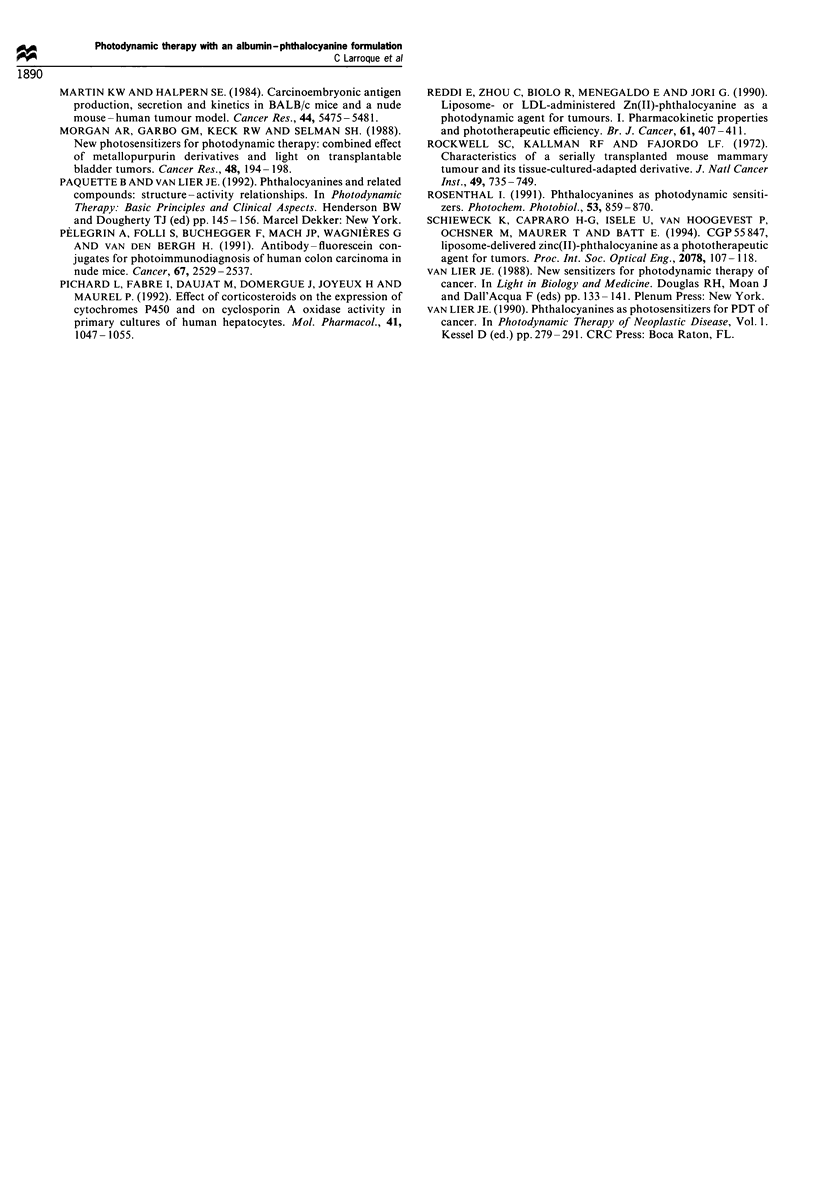# Serum albumin as a vehicle for zinc phthalocyanine: photodynamic activities in solid tumour models.

**DOI:** 10.1038/bjc.1996.649

**Published:** 1996-12

**Authors:** C. Larroque, A. Pelegrin, J. E. Van Lier

**Affiliations:** MRC Group in the Radiation Sciences, Faculty of Medicine, Université de Sherbrooke, Québec, Canada.

## Abstract

Zinc phthalocyanine (ZnPc) is a second-generation photosensitiser for the photodynamic therapy (PDT) of cancer. Unsubstituted ZnPc is, however, highly insoluble in most common solvents, and for clinical applications the material needs to be incorporated in liposomes. We report a simple, alternative procedure to formulate ZnPc through non-covalent binding to bovine serum albumin (BSA). Intravenous administration of ZnPc-BSA preparations, at a molar ratio of 11:1 and at a ZnPc dose equivalent to 0.5 mol kg-1, to tumour-bearing mice followed 24 h later by PDT was shown to provide tumour control in two different models, the EMT-6 tumour in Balb/c mice and the human colon T380 carcinoma in nude mice. Analysis of serum fractions from treated animals showed that ZnPc readily redistributes over the serum high-density lipoprotein (HDL) fraction. We also demonstrated the absence of hepatic toxicity of the ZnPc-BSA preparation by monitoring the hepatic cytochrome P450 activity in treated animals and the viability of human cultured hepatocytes.


British Journal of Cancer (1996) 74, 1886-1890
© 1996 Stockton Press All rights reserved 0007-0920/96 $12.00

### Serum albumin as a vehicle for zinc phthalocyanine: photodynamic activities in solid tumour models

C Larroque[1], A Pelegrin[2] and JE Van Lier[1]

[1]*MRC Group in the Radiation Sciences, Faculty of Medicine, Université de Sherbrooke, Sherbrooke, Québec, J1H 5N4, Canada;* [2]*Service de Médecine Nucléaire, Centre Régional de Lutte contre le Cancer, Val D'Aurelle Paul Lamarque et Faculté de Médecine, 34298 Montpellier, France.*

**Summary** Zinc phthalocyanine (ZnPc) is a second-generation photosensitiser for the photodynamic therapy (PDT) of cancer. Unsubstituted ZnPc is, however, highly insoluble in most common solvents, and for clinical applications the material needs to be incorporated in liposomes. We report a simple, alternative procedure to formulate ZnPc through non-covalent binding to bovine serum albumin (BSA). Intravenous administration of ZnPc-BSA preparations, at a molar ratio of 11:1 and at a ZnPc dose equivalent to 0.5 mol $kg^{-1}$, to tumour-bearing mice followed 24 h later by PDT was shown to provide tumour control in two different models, the EMT-6 tumour in Balb/c mice and the human colon T380 carcinoma in nude mice. Analysis of serum fractions from treated animals showed that ZnPc readily redistributes over the serum high-density lipoprotein (HDL) fraction. We also demonstrated the absence of hepatic toxicity of the ZnPc-BSA preparation by monitoring the hepatic cytochrome P450 activiy in treated animals and the viability of human cultured hepatocytes.

**Keywords:** photodynamic therapy; zinc phthalocyanine; human tumour xenograph; liver toxicity

Fluorescence properties and preferential retention of dyes in malignant tissues allow the clinical diagnosis of various human neoplasms (Dougherty *et al.*, 1978). If the photo-excited dyes are capable of *in situ* energy or electron transfer, they can also be used as sensitisers for the photodynamic therapy (PDT) of tumours. The most widely studied dye for PDT, Photofrin (PII), consists of a mixture of haematoporphyrin derivatives, and this preparation has been approved for clinical use (Levy, 1995). However, the relatively low absorbance above 600 nm (at which tissues provide optimal transparency) and some pharmacodynamic drawbacks have led to the development of new dyes for PDT (Morgan *et al.*, 1988; van Lier, 1988). Among them, the phthalocyanines (Pc) have advantageous photophysical properties and numerous studies have been conducted to demonstrate the potential of these molecules for PDT (van Lier, 1990; Rosenthal, 1991). Low solubility of the non-substituted metallo Pcs in biological fluids prompted chemical modifications to decrease their hydrophobicity (Ali *et al.*, 1988; Chan *et al.*, 1990). Albeit such changes may result in a decrease in their *in vivo* photoactivity, mono- and di-sulphonated derivatives are capable of cell membrane penetration resulting in increased potency to inflict direct cell kill during PDT (Brasseur *et al.*, 1988; Paquette and van Lier, 1992; Margaron *et al.*, 1996). Among the non-substituted Pcs, the zinc complex (ZnPc) has been studied in great detail (Ginevra *et al.*, 1990; Reddi *et al.*, 1990). Owing to its extreme insolubility in most common solvents, ZnPc needs to be incorporated in liposomes, and a proprietary formulation has been proposed for clinical use (Schieweck *et al.*, 1994).

In this study, we evaluate the PDT potential of the readily available, unsubstituted ZnPc solubilised with bovine serum albumin (BSA) and compare its PDT potency with that of two sulphonated ZnPcS derivatives. Photodynamic properties of the latter dyes have previously been reported in mice bearing the sarcoma type EMT-6 mouse mammary tumour (Paquette and van Lier, 1992), and we used the same model in the present study. In addition, we evaluated tumour control by ZnPc-BSA-PDT in the human colonic carcinoma T380 implanted in nude mice. The dark toxicity and possible effects of this preparation on mouse liver-detoxifying enzymes, such as cytochromes P450 and glutathione transferase, is evaluated. Behaviour of human cultured hepatocytes exposed to the ZnPc-BSA preparation is also reported.

Correspondence: C Larroque, INSERM, Unité 128, Route de Mende, BP 5051, 34033 Montpellier Cedex, France
Received 11 March 1996; revised 11 July 1996; accepted 16 July 1996

### Materials and methods

### *Animals and tumour models*

Mice were maintained and handled in accordance with the recommendations of a local animal care committee. The animals were allowed free access to water and food throughout the course of the experiment. The EMT6 mouse mammary tumour cell line was obtained from Dr C-W Lin (Massachusetts General Hospital, Boston) and maintained according to an established protocol (Rockwell *et al.*, 1972). Tumours were implanted in Balb/c mice on each hind thigh by intradermal injection of $2 \times 10^5$ EMT6 cells suspended in 50 $\mu$l of Waymouth growth medium. The human colon carcinoma T380 (Martin and Halpern, 1984) was serially transplanted subcutaneously into the right flank of 7- to 9-week-old female Swiss nu/nu mice (Iffra Credo, l'Arbresle, France). After 2 weeks, the mice were randomised to different treatment and control groups. Both models were used for PDT when tumours reached at least 8-10 mm in diameter. The tumour volume on nude mice was evaluated by measuring the tumour diameters (three dimensions) and applying the formula volume $= r1 \times r2 \times r3 \times 4\pi/3$ (r = radius).

### *Zinc phthalocyanine-albumin preparation*

Zinc phthalocyanine (Aldrich 34,116-9) was prepared as a saturated (1.7 mM) dimethylformamide (DMF) solution. This solution was added dropwise to a 1 mg $ml^{-1}$ bovine serum albumin (BSA) preparation in phosphate buffer (50 mM, pH 7.4) under gentle stirring. The final DMF concentration did not exceed 5%. Solid ammonium sulphate (0.625 g $ml^{-1}$, 85% saturation) was added to the mixture, which was then stirred at 4°C for 10 min and centrifuged at 10 000 *g* for 10 min. The clear supernatant was discarded and the coloured pellet was redissolved in phosphate buffer. This procedure was repeated until the ZnPc-BSA ratio remained stable. The final preparation was dialysed overnight against PBS to remove the excess of ammonium sulphate and used immediately or lyophylised. The lyophylised ZnPc-BSA preparation can be reconstituted in PBS and this solution was shown to be stable for several weeks at 4°C. ZnPc-BSA ratios were measured after a 100-fold dilution in DMF (ZnPc: $\lambda_{max} = 678$ nm, $\varepsilon = 2.45 \times 10^5$ $M^{-1}$ $cm^{-1}$).

*Phototherapy*

Either Balb/c or nude tumour-bearing mice were given an intravenous injection of the dye preparation via the lateral tail vein (maximal volume 0.2 ml). Phototherapy was applied 24 h after dye administration. EMT-6 or T380 tumour-bearing mice were anaesthetised with an intraperitoneal injection of tribromoethanol (230 mg $kg^{-1}$ in PBS, 10% ethanol). The tumours were centred under the red spot (diameter 1-1.5 mm) of a light source consisting of a focalised Xenon Cermax LX-300 F (ILC Technology, Sunnyvale, CA, USA) fitted with a 10 cm long water cell to remove infrared radiation and a 676 nm interference filter (bandpass $\pm$ 10 nm) (Optometrics, Leeds, UK). Illumination times were adjusted to deliver the desired light dose, as measured with a Photon Power Meter (Spectra-Physics Model 404). No hyperthermia was observed under these conditions. Response of the EMT-6 tumour was assessed by observation of the tumour necrosis followed by the absence of palpable tumour within 3 weeks after the treatment. In the case of the T380 tumours, the measurement of the tumour volume over a 2 month period was used to assess tumour response.

*Fractionation of serum proteins*

Balb/c mice blood was collected 24 h after injection of the ZnPc-BSA (11 : 1) preparation (2 $\mu$mol of ZnPc per kg) and the serum was separated after a short centrifugation. The various protein components were then separated on a potassium bromide density gradient in a Beckman VTi 65-2 rotor operating at 60 000 *g* for 2 h (Chung *et al*., 1986). The potassium bromide concentration was adjusted in order to obtain a final gradient density between 1.06 and 1.22. Fractions were then recovered from the top of the tube. The density of each fraction was monitored using a thermostated PAAR DMA 40 Digital Density Meter (Anton Parr, Graz, Austria) and ZnPc fluorescence was measured on an Aminco-Bowman spectrofluorimeter ($\lambda_{ex} = 646$ nm, $\lambda_{em} = 680$ nm, 5 nm bandpass) after calibration of the apparatus with successive dilutions of ZnPc in mouse serum. The nature of the proteins in each fraction was verified by polyacrylamide gel electrophoresis. The amont of total protein was estimated using the BCA method (Pierce, Rockford, MA) and bromocresol green was used to quantify albumin specifically in each fraction (Doumas *et al*., 1971).

*Liver detoxification*

Balb/c mice were injected intraperitoneally with a ZnPc-BSA (11 : 1) preparation (ZnPc dose of 2 $\mu$mol $kg^{-1}$) over a 4 day period. On day 5, the animals were sacrificed, livers removed and the microsomal and cytosolic fraction prepared. Progesterone is a good substrate for hepatic cytochrome P-450, and the pattern of metabolites is indicative of the concentration and nature of the various hydroxylases. Microsomal metabolic profiles of progesterone were prepared as previously described (Larroque *et al*., 1989).

*Enzymatic assays and human hepatocyte handling*

Microsomes and cytosol from treated or untreated mice were prepared by successive centrifugation as previously described (Lu and Levin, 1972). The microsomal cytochrome P-450 activity was tested using progesterone as substrate, and the metabolite composition was analysed after thin-layer chromatography and liquid scintillation counting (Larroque *et al*., 1989). Total glutathione-*S*-transferase activity was assayed on the cytosolic fraction with 1-chloro-2,4-dinitrobenzene as substrate (Habig *et al*., 1974). Human hepatocytes were prepared from hepatic lobectomies (as approved by the French National Ethics Committee), and cultured in a serum-free medium as previously described (Diaz *et al*., 1990). The overall metabolic integrity of hepatocytes in culture was assessed by measuring the rate of *de novo* protein synthesis in 24 h (Pichard *et al*., 1992), after the cells have been incubated in the dark for 6 days with various concentrations of the ZnPc-BSA preparation. All of the chemicals used in this study were of the highest purity commercially available.

### Results

Figure 1 shows the electronic spectrum of a ZnPc-BSA preparation. Superimposed is the transmission of the red light source. The relatively short bandpass from the 676 nm interference filter limits unwanted interactions with endogenous porphyrins. For a typical ZnPc-BSA preparation the dye content was 11 mol of ZnPc per mol of BSA. A lower ratio diminished the PDT potency, whereas a higher loading ratio did not improve tumour response.

*Photodynamic therapy*

The PDT efficacy of ZnPc-BSA was first compared with the known activities of the mono- and tetra-sulphonated $ZnPcS_1$ and $ZnPcS_4$ in Balb/c mice bearing EMT-6 mammary tumours. Tumour responses, after irradiation with red light ($\lambda = 676$ nm, 400 J $cm^{-2}$, 100 mW $cm^{-2}$) 24 h after injection of the various photosensitiser preparations, are summarised in Table I. These data show that tumour control can be achieved under these conditions with ZnPc-BSA at a ZnPc dose level of 0.5 $\mu$mol $kg^{-1}$. These results demonstrate that absorption of commercially available ZnPc to serum albumin provide a dye preparation with similar PDT efficacy to the synthetic, water-soluble $ZnPcS_1$ preparation. These data

**Figure 1** Electronic absorption spectrum of a ZnPc-BSA (11 : 1) preparation (—) and transmission of the interference filter used for the phototherapy (- - -).

**Table I** PDT of EMT6 tumours grafted on Balb/c mice

| Dye preparation | Dose ($\mu$mol ZnPc per kg) | Animals included in the series | Tumour evolution[b] after 24 h | 96 h | 1 week | 3 weeks |
|---|---|---|---|---|---|---|
| ZnPc-BSA | 0.1 | 6 | B | C | D | A |
| ZnPc-BSA | 0.5 | 18 | B | C | E | E |
| ZnPc-BSA | 1.0 | 12 | B | C | E | E |
| Free-$ZnPcS_1$ | 0.5 | 12 | B | C | E | E |
| Free-$ZnPcS_4$ | 1.0 | 6 | A | A | A | A |

[a] ZnPc-BSA (ratio 11 : 1); $S_1$, monosulphonate; $S_4$, tetrasulphonate. [b] A, no effect; B, black oedema; C, flat necrotic area; D, tumour regrowth; E, no palpable tumour.

prompted us to extend our evaluation to a human tumour model. The human colon carcinoma T380 grafted on nude mice was selected, since this type of cancer is clinically readily accessible for PDT, and this model has been used in many radiotherapy and radioimmunotherapy studies (Martin and Halpern, 1984; Buchegger *et al.*, 1990). The particular physiology of nude mice (especially transparency of the skin) obliged us to use a lower light dose to avoid liver dysfunction after illumination. A maximum light dose of 200 J cm$^{-2}$ with a fluence rate of 30 mW cm$^{-2}$ was selected for these experiments; the administered ZnPc dose being arbitrarily adjusted to 2 $\mu$mol kg$^{-1}$. Mice that received the ZnPc–BSA preparation and light were compared with untreated mice as well as with mice that received dye or light only. In all mice that received the complete PDT treatment (dye and illumination), tumour damage was clearly visible within 8 h after irradiation leading to a black oedema and blood leakage around the tumour, which became less adherent to the muscle and the skin of the animal. In the following 4 days, the oedema disappeared leading instead to a necrotic area for the next 5–7 days. Subsequently, the treated animals were apparently tumour free up to 25 days after the PDT. None of these responses were observed in the control animals, which showed an exponential tumour growth to an average tumour size of 500 mg on day 24 after inoculation (Figure 2). In the treated group, the 500 mg tumour size was observed only on day 49 after the tumour implant. The regrowth of the tumour in the treated mice that were apparently tumour free could be attributed to cells which did not receive the complete light dose, i.e. deeper-seated cells or cells outside the light spot.

### *Serum distribution of ZnPc*

Figure 3 depicts the ZnPc fluorescence, the albumin content and the density of each 500 $\mu$l fraction collected after centrifugation of serum from dye-treated animals in a potassium bromide density gradient. The albumin-rich fraction is at the bottom of the tube, whereas the ZnPc fluorescence is associated with a protein fraction of a density between 1.09 and 1.13. This density range is compatible with that of high-density lipoproteins (HDLs) (Gotto *et al.*, 1986). No fluorescence was detected in lighter or heavier fractions revealing that ZnPc did not associate with lipoproteins of low density (chylomicrons, LDL or IDL) and that complete transfer from albumin to HDL had occurred. The identity of the protein fraction carrying ZnPc as HDL was confirmed by a polyacrylamide gel electrophoresis (data not shown), which permits the detection of 18 000 to 22 000 Da fragments comparable in molecular weight to ApoA-II or ApoD from

**Figure 2** Evolution of the volume (mean and standard deviation) of T380 experimental tumours grafted on nude mice receiving on day 10 after the graft: no treatment (○), light alone (△), ZnPc–BSA (2 $\mu$mol ZnPc per kg) alone (□), or ZnPc–BSA (2 $\mu$mol ZnPc per kg) plus light (**X**).

**Figure 3** Fractionation of serum proteins by centrifugation 24 h after the mice were injected with a preparation of ZnPc–BSA. The fractions were collected from the top to the bottom of the centrifuge tube. The density (- - -), the albumin concentration ( ) and the phthalocyanine fluorescence ( ) were assayed in each fraction.

high-density lipoproteins (Gotto *et al.*, 1986). This ZnPc distribution profile contrasts with the pattern observed by Ginevra *et al.* (1990) using ZnPc incorporated in a liposomal vehicle. It is evident that the dye formulation greatly influences serum distribution of the ZnPc. In view of these results, we also measured PDT response after ZnPc administration as a mouse HDL–ZnPc complex and found that tumour response was in all respects similar to that observed with the ZnPc–BSA preparation (data not shown).

### *Effects of ZnPc–BSA on liver detoxifying systems*

Progesterone metabolic profiles and cytochrome P-450 content of hepatic microsomes from treated and untreated animals were compared. No significant difference could be detected, either on the cytochrome P-450 content or on the nature or amount of progesterone metabolites produced. Similarly, no effect was detectable on the glutathione transferase activity in the hepatic cytosolic fractions derived from the various animals. An enzyme activity of 35$\pm$4 nmol of chloro 2,6-dinitrobenzene conjugated per min and per mg of cytosolic proteins was observed for all cytosols tested, both from treated or untreated mice.

**Figure 4** Effect of various concentrations of ZnPc–BSA preparation on human cultured hepatocytes. The dark toxicity is measured as the influence of the dye preparation on the incorporation of tritiated leucine in *de novo* synthesised proteins after 6 days incubation in the dark.

*Effect of ZnPc-BSA on human cultured hepatocytes*

As stated, phthalocyanines are easily trapped by the liver. The capacity of human hepatocytes to synthesise proteins after 6 days of incubation with various concentrations of ZnPc-BSA is seen in Figure 4. The lowest concentration used (e.g. 6 $\mu$M of BSA-bound ZnPc) corresponds to the maximum ZnPc concentration in the serum of mice that received ZnPc-BSA equivalent to a ZnPc dose of 1 $\mu mol\ kg^{-1}$. As can be seen from Figure 4, toxicity only becomes evident at ZnPc concentrations exceeding 30 $\mu$M. This observation is in line with our other findings and confirms the relatively low toxicity of the ZnPc-BSA preparation.

### Discussion

The delivery of hydrophobic dyes to tumour cells has been proposed to proceed via a transport by lipoproteins (Ginevra *et al.*, 1990). The present study demonstrates that the BSA-delivered ZnPc readily redistributes from the carrier protein to the serum lipoproteins, particularly the HDL fraction. Thus, the *in vivo* PDT effect following ZnPc-BSA administration is likely to involve the same mechanism as that observed with similar dyes delivered via liposomal preparations (Reddi *et al.*, 1990). Compared with the latter, ZnPc loading on albumin is a relatively simple procedure (Schieweck *et al.*, 1994). To avoid antigenecity problems, it is evident that the same procedure can be used with human albumin preparations.

The PDT efficacy of the ZnPc-BSA is similar to that of the monosulphonated $ZnPcS_1$. However, the latter preparation requires extensive chromatographic purification and consists of a mixture of positional isomers (Ali *et al.*, 1988). Accordingly, the ZnPc-albumin formulation promises to be more suitable for scaling up to the quantities required in a clinical setting.

The liver is the principal site for the oxidation and detoxification of foreign materials, and the lipophilic phthalocyanines are known to be largely retained by the liver. Previous *in vitro* studies on isolated liver microsomes demonstrated the deleterious effect of phthalocyanine on these structures (Agarwal *et al.*, 1992). To mimic more closely the photodynamic protocol, we studied the *in vivo* effect of ZnPc-BSA on some essential liver functions. Finally, we showed for the first time the lack of hepatic dark toxicity of ZnPc, even at relative high dye concentrations. However, the apparent accumulation of the dye in the liver remains a problem, necessitating the use of lower light doses in our nude mice model. Although the BSA vehicle allowed the biodistribution of the dye throughout the whole animal, increased tumour selectivity would obviously be desirable. Covalent coupling of other dyes (fluorescein, indocyanin) to a monoclonal antibody directed against tumour cells in nude mice bearing colon or squamous cell carcinomas has been demonstrated (Folli *et al.*, 1992, 1994; Pèlegrin *et al.*, 1991), and a similar approach for ZnPc targeting may further enhance tumour specificity.

**Acknowledgements**
The authors are grateful to the Medical Research Council of Canada and la Ligue de Recherche contre le Cancer for support of this work, and to Dr P Maurel for advice on human hepatocyte culture. CL acknowledges a fellowship from the Fonds de la Recherche en Santé du Québec.

### References

AGARWAL R, ZAIDI SIA, ATHAR M, BICKERS DR AND MUKTAR H. (1992). Photodynamic effects of chloroaluminium phthalocyanine tetrasulfonate are mediated by singlet oxygen: *in vivo* and *in vitro* studies utilizing hepatic microsomes as a model membrane source. *Arch. Biochem. Biophys.*, **294**, 30-37.

ALI H, LANGLOIS R, WAGNER JR, BRASSEUR N, PAQUETTE B AND VAN LIER JE. (1988). Biological activities of phthalocyanines-X. Syntheses and analyses of sulfonated phthalocyanines. *Photochem. Photobiol.*, **47**, 713-719.

BRASSEUR N, ALI H, LANGLOIS R AND VAN LIER JE. (1988). Biological activities of phthalocyanines-IX. Photosensitization of V-79 Chinese hamster cells and EMT-6 mouse mammary tumors in mice with sulfonated phthalocyanines. *Photochem. Photobiol.*, **47**, 705-711.

BUCHEGGER G, PÈLEGRIN A, DELALOYE B, BISCHOF-DELALOYE A AND MACH JP. (1990). $^{131}$I labeled $F(ab')_2$ fragments are more efficient and less toxic than intact anti-CEA antibodies in radioimmunotherapy of large human colon carcinoma grafted in nude mice. *J. Nucl. Med.*, **31**, 1035-1044.

CHAN WS, MARSHALL JF, SVENSEN R, BEDWELL J AND HART IR. (1990). Effect of sulfonation on the cell and tissue distribution of the photosensitizer aluminium phthalocyanine. *Cancer Res.*, **50**, 4533-4538.

CHUNG BH, SEGREST JP, RAY MJ, BRUNZELL JD, HOKANSON JE, KRAUSS RM, BEAUDRIE K AND CONE JT. (1986). Single vertical spin density gradient ultracentrifugation. *Methods Enzymol.*, **128**, 181-209.

DIAZ D, FABRE I, DAUJAT M, SAINT-AUBERT B, BORIES P, MICHEL H AND MAUREL P. (1990). Omeprazole is an aryl hydrocarbon-like inducer of human hepatic cytochrome P450. *Gastroenterology*, **99**, 737-747.

DOUGHERTY TJ, GOLDFARB A, WEISHAUPT KR, BOYLE DG AND MITTLEMAN A. (1978). Photoradiation therapy for the treatment of malignant tumors. *Cancer Res.*, **38**, 2628-2635.

DOUMAS BT, WATSON WA AND BIGGS HG. (1971). Albumin standards and the measurement of serum albumin with bromocresol green. *Clin. Chim. Acta*, **31**, 87-96.

FOLLI S, WAGNIÈRES G, PÈLEGRIN A, CALMES JM, BRAICHOTTE D, BUCHEGGER F, CHALANDON Y, HARDMAN N, HEUSSER C, GIVEL JC, CHAPUIS G, CHÂTELAIN A, VAN DEN BERG H AND MACH JP. (1992). Immunophotodiagnosis of colon carcinomas in patients injected with fluoresceinated chimeric antibodies against carcinoembryonic antigen. *Proc. Natl Acad. Sci. USA*, **89**, 7973-7977.

FOLLI S, WESTERMANN P, BRAICHOTTE D, PÈLEGRIN A, WAGNIÈRES G, VAN DEN BERG H AND MACH JP. (1994). Antibody-indocyanine conjugates for the immunophotodetection of a human squamous cell carcinoma in nude mice. *Cancer Res.*, **54**, 2643-2649.

GINEVRA F, BIFFANTI S, PAGNAN A, BIOLO R, REDDI E AND JORI G. (1990). Delivery of the tumour photosensitizer zinc(II)-phthalocyanine to serum proteins by different liposomes: studies *in vitro* and *in vivo*. *Cancer Lett.*, **49**, 59-65.

GOTTO AM, POWNALL HJ AND HAVEL JH. (1986). Introduction to the plasma lipoproteins. *Methods Enzymol.*, **128**, 3-41.

HABIG WH, PABIST MJ AND JAKOBY WB. (1974). Glutathion transferase: the first enzymatic step in mercapturic acid formation. *J. Biol. Chem.*, **249**, 7130-7139.

LARROQUE C, LANGE R, MAUREL P, LANGLOIS R AND VAN LIER JE. (1989). Rat liver microsomal progesterone metabolism: evidence for differential troleandomycin and pregnenolone 16a-carbonitrile inductive effects in the cytochrome P-450 III family. *J. Steroid Biochem.*, **33**, 277-286.

LEVY JG. (1995). Photodynamic therapy. *Trends in Biotechnol.*, **13**, 14-18.

LU AYH AND LEVIN W. (1972). Partial purification of cytochromes P-450 and P-448 from rat liver microsomes. *Biochem. Biophys. Res. Commun.*, **46**, 253-256.

MARGARON P, MADARNAS P, OUELLET R AND VAN LIER JE. (1996). Biological activities of phthalocyanines-XVII. Histopathologic evidence for different mechanisms of EMT-6 tumor necrosis induced by photodynamic therapy with disulphonated aluminium phthalocyanine or Photofrin. *Anticancer Res.*, **16**, 613-629.

MARTIN KW AND HALPERN SE. (1984). Carcinoembryonic antigen production, secretion and kinetics in BALB/c mice and a nude mouse-human tumour model. *Cancer Res.*, **44,** 5475-5481.

MORGAN AR, GARBO GM, KECK RW AND SELMAN SH. (1988). New photosensitizers for photodynamic therapy: combined effect of metallopurpurin derivatives and light on transplantable bladder tumors. *Cancer Res.*, **48,** 194-198.

PAQUETTE B AND VAN LIER JE. (1992). Phthalocyanines and related compounds: structure-activity relationships. In *Photodynamic Therapy: Basic Principles and Clinical Aspects.* Henderson BW and Dougherty TJ (ed) pp. 145-156. Marcel Dekker: New York.

PÈLEGRIN A, FOLLI S, BUCHEGGER F, MACH JP, WAGNIÈRES G AND VAN DEN BERGH H. (1991). Antibody-fluorescein conjugates for photoimmunodiagnosis of human colon carcinoma in nude mice. *Cancer*, **67,** 2529-2537.

PICHARD L, FABRE I, DAUJAT M, DOMERGUE J, JOYEUX H AND MAUREL P. (1992). Effect of corticosteroids on the expression of cytochromes P450 and on cyclosporin A oxidase activity in primary cultures of human hepatocytes. *Mol. Pharmacol.*, **41,** 1047-1055.

REDDI E, ZHOU C, BIOLO R, MENEGALDO E AND JORI G. (1990). Liposome- or LDL-administered Zn(II)-phthalocyanine as a photodynamic agent for tumours. I. Pharmacokinetic properties and phototherapeutic efficiency. *Br. J. Cancer*, **61,** 407-411.

ROCKWELL SC, KALLMAN RF AND FAJORDO LF. (1972). Characteristics of a serially transplanted mouse mammary tumour and its tissue-cultured-adapted derivative. *J. Natl Cancer Inst.*, **49,** 735-749.

ROSENTHAL I. (1991). Phthalocyanines as photodynamic sensitizers. *Photochem. Photobiol.*, **53,** 859-870.

SCHIEWECK K, CAPRARO H-G, ISELE U, VAN HOOGEVEST P, OCHSNER M, MAURER T AND BATT E. (1994). CGP 55 847, liposome-delivered zinc(II)-phthalocyanine as a phototherapeutic agent for tumors. *Proc. Int. Soc. Optical Eng.*, **2078,** 107-118.

VAN LIER JE. (1988). New sensitizers for photodynamic therapy of cancer. In *Light in Biology and Medicine.* Douglas RH, Moan J and Dall'Acqua F (eds) pp. 133-141. Plenum Press: New York.

VAN LIER JE. (1990). Phthalocyanines as photosensitizers for PDT of cancer. In *Photodynamic Therapy of Neoplastic Disease*, Vol. 1. Kessel D (ed.) pp. 279-291. CRC Press: Boca Raton, FL.